# The contribution of specific non-communicable diseases to the achievement of the Sustainable Development Goal 3.4 in Peru

**DOI:** 10.1371/journal.pone.0240494

**Published:** 2020-10-12

**Authors:** Rodrigo M. Carrillo-Larco, James E. Bennett, Mariachiara Di Cesare, Edward W. Gregg, Antonio Bernabe-Ortiz

**Affiliations:** 1 Department of Epidemiology and Biostatistics, School of Public Health, Imperial College London, London, United Kingdom; 2 CRONICAS Centre of Excellence in Chronic Diseases, Universidad Peruana Cayetano Heredia, Lima, Peru; 3 Middlesex University, London, United Kingdom; 4 Universidad Científica del Sur, Lima, Peru; Chiang Mai University Faculty of Medicine, THAILAND

## Abstract

**Background:**

Non-communicable diseases (NCDs) have received political attention and commitment, yet surveillance is needed to measure progress and set priorities. Building on global estimates suggesting that Peru is not on target to meet the Sustainable Development Goal 3.4, we estimated the contribution of various NCDs to the change in unconditional probability of dying from NCDs in 25 regions in Peru.

**Methods:**

Using national death registries and census data, we estimated the unconditional probability of dying between ages 30 and 69 from any and from each of the following NCDs: cardiovascular, cancer, diabetes, chronic respiratory diseases and chronic kidney disease. We estimated the contribution of each NCD to the change in the unconditional probability of dying from any of these NCDs between 2006 and 2016.

**Results:**

The overall unconditional probability of dying improved for men (21.4%) and women (23.3%). Cancer accounted for 10.9% in men and 13.7% in women of the overall reduction; cardiovascular diseases also contributed substantially: 11.3% in men) and 9.8% in women. Consistently in men and women and across regions, diabetes moved in the opposite direction of the overall reduction in the unconditional probability of dying from any selected NCD. Diabetes contributed a rise in the unconditional probability of 3.6% in men and 2.1% in women.

**Conclusions:**

Although the unconditional probability of dying from any selected NCD has decreased, diabetes would prevent Peru from meeting international targets. Policies are needed to prevent diabetes and to strengthen healthcare to avoid diabetes-related complications and delay mortality.

## Introduction

The global burden of non-communicable diseases (NCDs) has garnered increasing attention because of their impact on individuals, health systems, and national economies alike. This became clear in 2011 at the first United Nations high-level meeting on non-communicable disease prevention and control [1]. After this event, the World Health Organization established nine voluntary goals, including to achieve a 25% reduction in the overall mortality from key NCDs: cardiovascular diseases, cancer, chronic respiratory diseases and diabetes [2]. In 2015 the United Nations issued the Sustainable Development Goals, which included health-related targets of reducing premature mortality from NCDs by 2030, with emphasis on the same four diseases [3]. In 2018, the United Nations hosted the third high-level meeting on NCDs [4], where progress on the original targets were evaluated and evidence signalled that many low- and middle-income countries may not meet the targets on time [5]. However, these country-level estimates may not reflect the full reality within each country, particularly in low- and middle-income countries where socio-economic inequalities play a relevant role in the distribution of cardio-metabolic risk factors and NCDs mortality [6]. Therefore, to complement the global evidence and to also strengthen local knowledge to inform policies and interventions, we analysed the unconditional probability of dying from selected NCDs in 25 regions in Peru. We examined the positive or negative contribution of five specific chronic conditions (i.e. cardiovascular disease, cancer, chronic respiratory disease, diabetes, and chronic kidney disease) to the Sustainable Development Goal 3.4 of reducing NCDs-related premature mortality; of note, this goal seeks a one-third reduction of premature mortality from selected non-communicable diseases by 2030 [3]. This information, at national and sub-national level may aid in the prioritization of resources to meet international targets to reduce the burden of NCDs in Peru.

## Materials and methods

**Fig 1:**
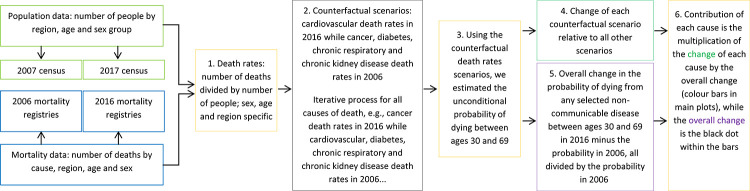
Summary of methods.

Using national death registries and census data we computed the unconditional probability of dying from any and each selected NCD in 25 regions in Peru [7]; also, we estimated the contribution of the selected NCDs to understand which followed a favourable trend and which moved in an opposite direction the change in the overall unconditional probability of dying from any of the selected NCDs between 2006 and 2016. Fig 1 presents a summary of the methods while Table 1 shows the formulas used in the analysis.

**Table 1:** Formulas used in the analysis.

Counterfactual scenarios (using cancer as example)	$\begin{array}{l} Death\;rate\;for\;cancer\;counterfactual\\ \;=Cardiovascular\;diseases\;death\;rate\;in\;2006+cancer\;death\;rate\;in\;2016+diabetes\;death\;rate\;in\;2006\\ \;+chronic\;respiratory\;diseases\;death\;rate\;in\;2006+chronic\;kidney\;disease\;death\;rate\;in\;2006 \end{array}$
Unconditional probability of dying between ages 30 to 69	$\begin{array}{l} Probability\;of\;death\;in\;each\;five-year\;age\;group\ldots 5qx\\ \;=\frac{5Mx*2.5}{1+5Mx*2.5\;}\ldots where\;5Mx\;is\;the\;mortality\;rate\;in\;five-year\;age\;group \end{array}$ $Unconditional\;probability\;of\;death\;from\;ages\;30\;to\;69\ldots 40q30=1-\prod_{x=30}^{65}\left(1-5qx\right)$
Change of each counterfactual scenario relative to all scenarios (using cancer as example)	$\frac{40q30[Death\;rate\;for\;cancer\;counterfactual]\;-\;40q30[Death\;rate\;for\;any\;selected\;NCD\;in\;2006]\;\;}{\begin{array}{c} 40q30[Death\;rate\;for\;cancer\;counterfactual]\;-\;40q30[Death\;rate\;for\;any\;selected\;NCD\;\;in\;2006]+\\ 40q30[Death\;rate\;for\;cardiovascular\;diseases\;counterfactual]\;-\;40q30[Death\;rate\;for\;any\;selected\;NCD\;\;in\;2006]+\\ 40q30[Death\;rate\;for\;diabetes\;counterfactual]\;-\;40q30[Death\;rate\;for\;any\;selected\;NCD\;\;in\;2006]+\\ 40q30[Death\;rate\;for\;chronic\;respiratory\;diseases\;counterfactual]\;-\;40q30[Death\;rate\;for\;any\;selected\;NCD\;\;in\;2006]+\\ 40q30[Death\;rate\;for\;chronic\;kidney\;disease\;counterfactual]\;-\;40q30[Death\;rate\;for\;any\;selected\;NCD\;\;in\;2006] \end{array}}$
Overall change in the probability of dying	$\frac{40q30[Death\;rate\;for\;any\;selected\;NCD\;in\;2016]-40q30[Death\;rate\;for\;any\;selected\;NCD\;in\;2006]}{40q30[Death\;rate\;for\;any\;selected\;NCD\;in\;2006]}$

### Study setting – Peru

**Fig 2:**
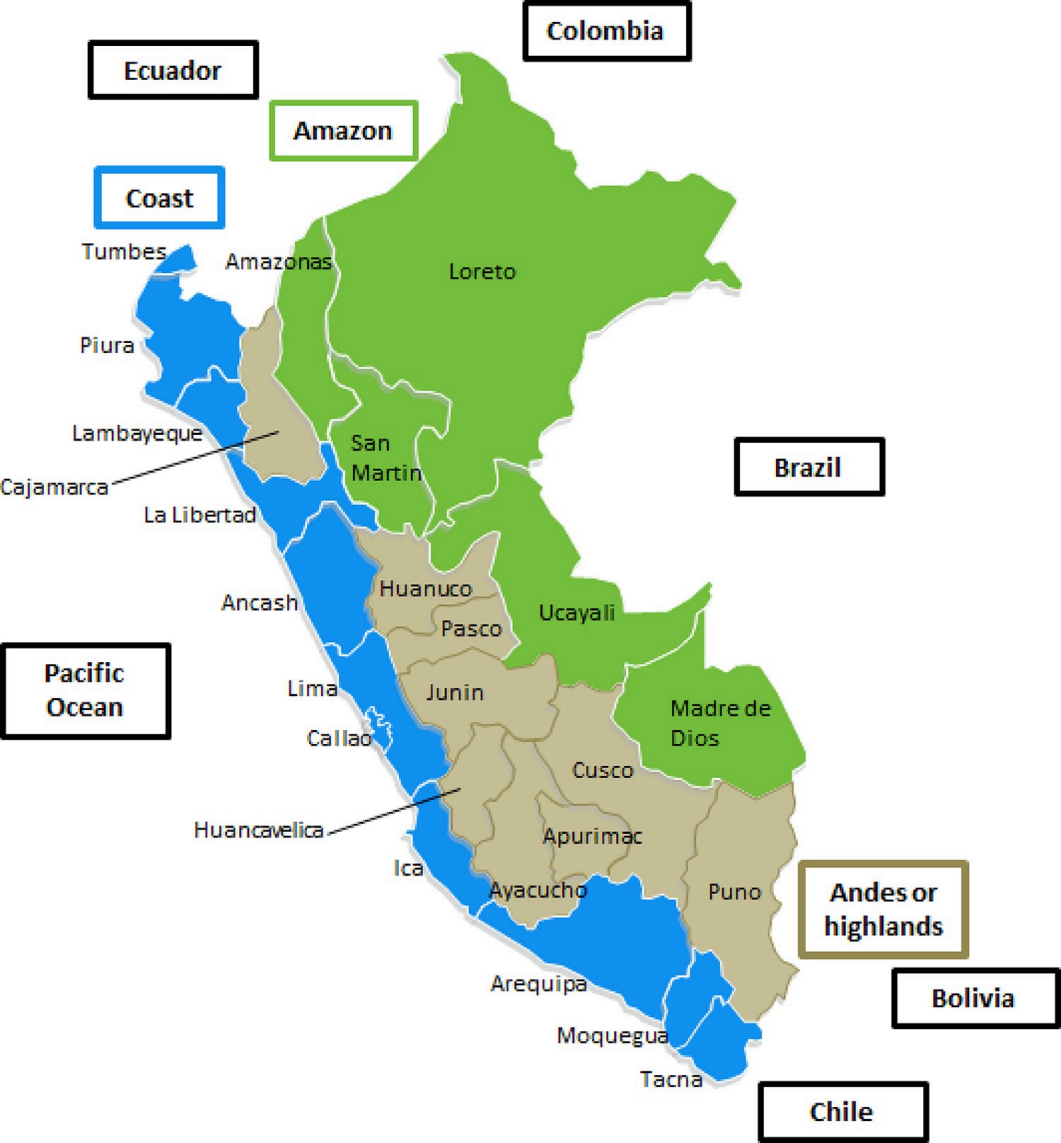
Map of Peru showing regions and macro-regions.

Peru is an upper-middle-income country (World Bank classification) in South America with a population of 32 million people, divided geo-politically into 25 regions (Fig 2) [8]. These regions can be grouped in three macro-regions: coast (high income regions at sea level), Andes or highlands (regions with low income and several at high altitude above the sea level), and the Amazon (middle-income regions mostly at sea level).

National estimates indicate that NCD deaths accounted for 70% of all deaths in 2017 [9]. The national mean body mass index (BMI) ranges between 26.4 kg/m^2^ and 27.3 kg/m^2^; also, 25.1% and 15.8% of the female and male adult Peruvian population are obese (BMI≥30 kg/m^2^) [10]. The prevalence of diabetes is higher in women than men (8.1% vs 7.2%) [11]. Conversely, the national prevalence of raised blood pressure is higher in men than in women (16.1% vs 11.2%) [12].

### Data sources

Data on cause of deaths by region, year, age and sex were requested and received from the Peruvian Ministry of Health mortality records in 2006 and 2016 (latest available year). Population data were from the national census in 2007 and 2017 (total population) [13, 14].

### Data preparation

Population data in 2007 [13] were combined with death records in 2006, whilst population data in 2017 [14] were combined with death records in 2016. This decision was taken because of data availability and to use the original census data instead of an extrapolation to match the exact same years (i.e. both population and mortality data in 2006).

The selected causes of death were cardiovascular diseases (International Classification of Diseases 10^th^ (ICD-10): I00-99), cancer (ICD-10: C00-97), diabetes (ICD-10: 10-14), chronic respiratory diseases (ICD-10: J30-98) and chronic kidney disease (ICD-10: N18). The first four are part of the World Health Organization Global Monitoring Framework for NCDs and the United Nations Sustainable Development Goal 3.4 [15, 16]. Chronic kidney disease was included because it shares risk factors with the other four causes and its burden is increasing in Peru [17, 18]. Death registries were adjusted to account for under-reporting and garbage codes following standard method. This method redistributes ill-defined (garbage) codes and under-reporting into the other categories. For this, it requires mortality data and life tables (account for the expected number of deaths), which were available from the Peruvian Institute of Statistics and informatics [19, 20]. The proportion of garbage codes ranged from 9% (Tacna) to 26% (Junin) in 2006, and from 12% (Tacna) to 79% (Ucayali); overall, the proportion was 18%, with not large differences between 2006 (18%) and 2016 (18%).

### Counterfactual scenarios

We developed five region-, age- and sex-specific counterfactual scenarios to determine the relative contribution of each condition to the total. Each counterfactual scenario included death rates in 2016 for one cause and death rates for the other causes in 2006. For example, in order to examine the impact of changes in cancer on the total, the counterfactual scenario included death rates of cancer in 2016, but death rates of cardiovascular disease, diabetes, chronic respiratory diseases, and chronic kidney disease in 2006 (Table 1).

### Unconditional probability

The unconditional probability refers to the probability of dying without any competing causes of death. The methods used are based on life tables informed by age-specific death rates (Table 1) [21]. Following the methodology used in other papers [5, 7, 21], we computed the unconditional probability of dying between ages 30 to 69 from any of the selected causes in 2006 and 2016. We used the above described counterfactual scenarios to estimate the unconditional probability of dying.

### Change estimation and relative contribution

With the outputs of the unconditional probability of dying formula, we estimated the overall change in the unconditional probability of dying between ages 30 to 69 from any of the selected causes of death between 2006 and 2016 (Table 1). In addition, we estimated the change for each counterfactual scenario in relation to all the scenarios (Table 1).

### Contribution of each selected cause of death

To estimate the contribution of each counterfactual scenario, and of each condition to the overall change in the probability of dying, we multiplied the change of each counterfactual scenario with the overall change of the unconditional probability. When the overall change was negative, it suggested that the overall probability has reduced (i.e., larger in 2006 than in 2016); conversely, when the overall change was positive, this suggested that the overall probability has increased (i.e., larger in 2016 than in 2006). When the contribution of a given selected diseases was in the same direction as the overall change (e.g., both negative), it meant that such condition has followed the trend of the overall change; conversely, when the contribution of a given cause moved in the opposite direction of the overall change (e.g., overall change was negative and the cause-specific was positive), it meant that such cause has stagnated the overall change.

### Life expectancy

Using life tables informed by the analysed population data, we estimated life expectancy in Peru and in each region (after the 2017 national census) [22]. We used the same methods as in previous publications [23], based on standard techniques [21]. Of note, this approach was used by the United Nations Population Division, the World Health Organization [24], the Human Mortality Database [25], and The Lancet Series on aging [26].

### Ethics

This work analysed publicly available de-identified data, thus of low risk for human subjects. No ethical approval was sought. The funder of the study had no role in study design, data collation, data analysis, data interpretation, or writing of the report. RMC-L had full access to all the data in the study and had final responsibility for the decision to submit for publication. All authors approved the submitted version.

## Results

### Selected causes of death – Crude proportions

In 2016, cardiovascular diseases were responsible for 16.0% of all deaths in men and 13.7% in women; these numbers in 2006 were 20.5% and 17.3%, respectively. In 2016, cancers were responsible for 21.9% and 39.6% of all deaths in men and women, respectively (24.7% and 42.3% in 2006). Of all deaths in 2016, diabetes was responsible for 5.8% in men and 6.4% in women, approximately twice that of the 2006 estimates (2.7% and 3.5%, respectively). Chronic respiratory diseases accounted for 4.3% and 4.1% of all deaths in men and women in 2016; with little variation from 2006 (4.6% and 3.9%). Finally, in 2016, chronic kidney disease accounted for 1.8% and 1.7% of all deaths in men and women, respectively; these numbers in 2006 were 2.5% and 2.8%.

### Overall unconditional probability of dying

**Fig 3:**
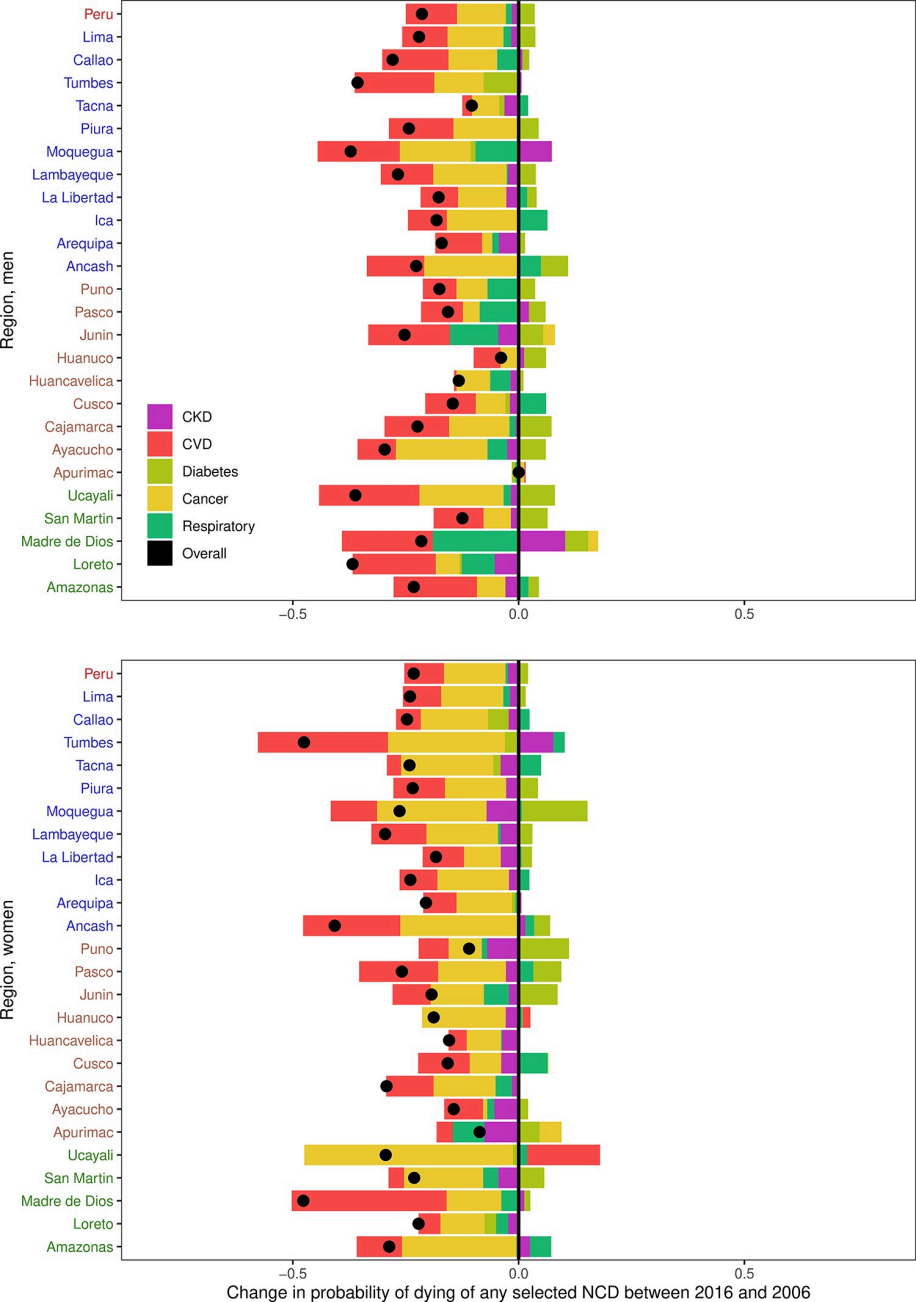
Change in the overall probability of dying and the contribution of each selected cause of death by gender and region in Peru. Regions in blue are in the Coast; regions in brown are in the Andes and regions in green are in the Amazon. A negative change in the overall probability of dying (black dot) means that the probability of dying has decreased between 2006 and 2016 (i.e., favourable change). Estimates are available in S1 Table.

Overall, between 2006 and 2016, the unconditional probability of dying between ages 30 and 69 from any of the selected NCDs has decreased for both men and women: a 21.4% and 23.2% reduction, respectively (Fig 3; S1 Table).

This reduction was largely promoted by cardiovascular diseases and cancer; the former contributed with ~10% (11.3% for men and 8.8% for women) and the latter ~12% (10.9% for men and 13.6% for women) of the total reduction (Fig 3; S1 Table). Chronic kidney disease (1.5% in men and 2.3% in women) and chronic respiratory diseases (1.3% in men and 0.6% in women) contributed smaller proportions to the reduction in the overall unconditional probability of dying (Fig 3; S1 Table). The contribution of diabetes, on the other hand, moved in the opposite direction of the overall reduction in the unconditional probability of dying, contributing an increase to the overall change of 3.6% in men and 2.1% in women (Fig 3; S1 Table). In other words, diabetes mortality is lagging the overall reduction in the unconditional probability of dying from the selected NCDs.

### Region-specific unconditional probability of dying

In all twenty-five regions, the overall unconditional probability of dying from the selected NCDs has decreased for men and women (Fig 3; S1 Table). Despite these promising numbers, between 2006 and 2016 the contribution of diabetes mortality to the overall unconditional probability of dying was mostly unfavourable (Fig 3; S1 Table). For men, in eighteen regions (4/5 in the Amazon, 7/9 in the Andes and 7/11 in the coast), the diabetes contribution moved to the opposite direction of the overall reduction in the unconditional probability of dying; this number of regions for women was sixteen (2/5 in the Amazon, 7/9 in the Andes and 7/11 in the coast). Where diabetes moved in the opposite direction of the overall trend, was largely in women from Moquegua (14.5%), though where diabetes positively contributed the most, i.e. followed the trend of the overall reduction, was in men from Tumbes (7.7%) (Fig 3; S1 Table).

**Fig 4:**
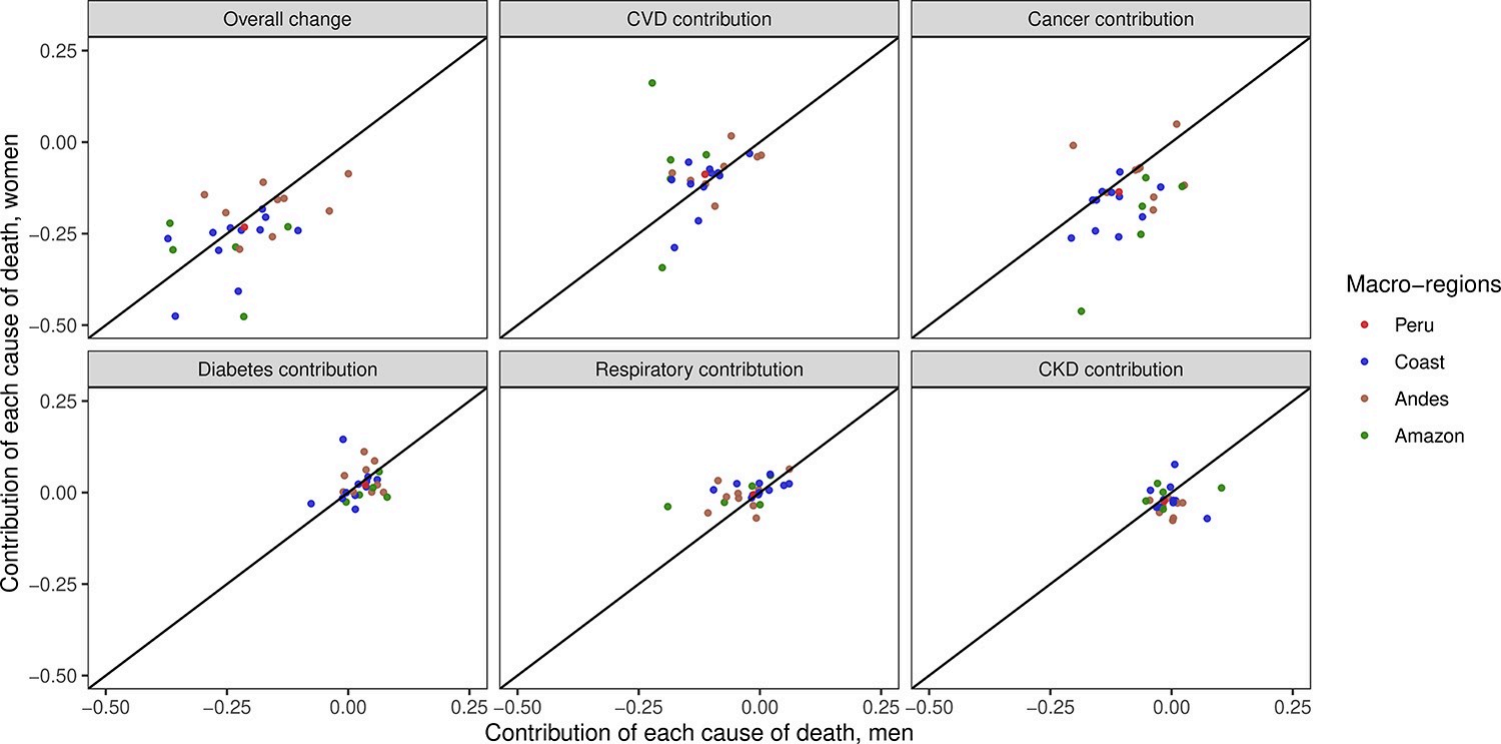
Overall change in the unconditional probability of dying between 2006 and 2016 and the contribution of the selected non-communicable diseases by macro-regions in Peru in women relative to men.

The contribution to the overall change of the unconditional probability of dying from the selected NCDs between 2006 and 2016 differed between men and women and by region (Fig 4; S1 Table). For example, regarding cancer and diabetes, in the Amazon the contribution was larger in men than in women (Fig 4; S1 Table). The contribution of chronic respiratory diseases and chronic kidney disease did not exhibit much variability across regions between men and women (Fig 4; S1 Table).

### Life expectancy and probability of dying

**Fig 5:**
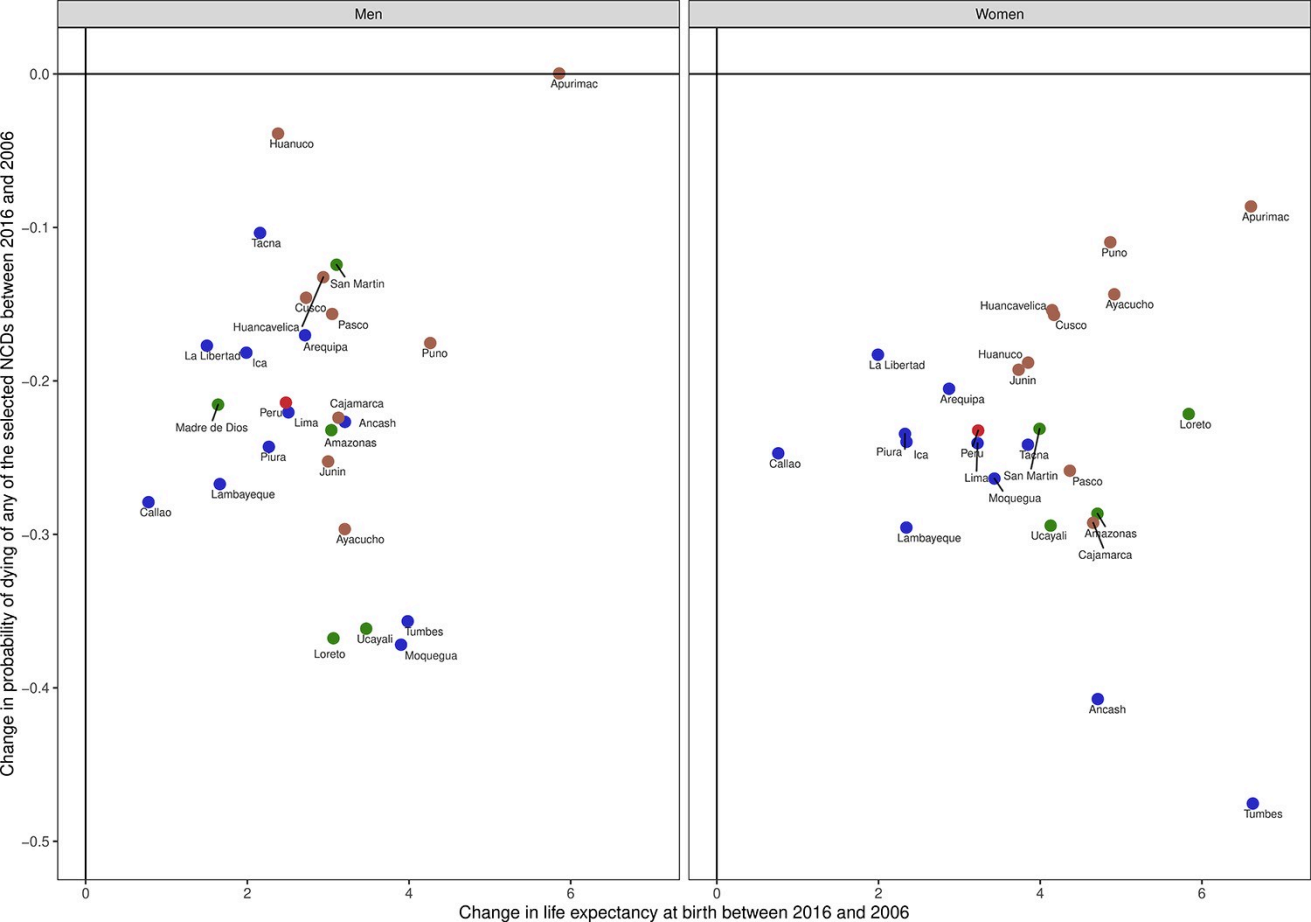
Change in probability of drying versus change in life expectancy by gender and region in Peru. Regions in blue are in the Coast; regions in brown are in the Andes and regions in green are in the Amazon. Madre de Dios is not included in the figure for women; life expectancy could not be estimated for this region because of very few or no deaths in the oldest age group. Estimates are Available in S2 Table.

The overall life expectancy in 2016 was 75 (73 in 2006) in men and 82 (78 in 2006) in women. In 2016 the lowest life expectancy in men was in Callao (Coast), Ica (Coast), Lambayeque (Coast), and La Libertad (Coast) with 74 years; the highest was in Apurimac (Andes), Moquegua (Coast), Pasco (Andes), Tumbes (Coast) and Ucayali (Amazon) with 77 years. In women in 2016, the lowest life expectancy was in Callao (Coast) and Ica (Coast) with 80 years, whereas the highest was in Tumbes with 85 years. All regions have improved both the overall unconditional probability of dying and life expectancy at birth (Fig 5; S2 Table).

## Discussion

According to the national estimate Peru is not on track to meet the Sustainable Development Goal 3.4 by 2030, yet is has improved in the last years [5]. Our work builds on this to explore the contribution of five key NCDs responsible for not meeting the target at national and subnational level. Across regions, in both men and women, diabetes represents a key barrier to the achievement of global goals aiming to reduce premature mortality from NCDs [3].

Over the period of analysis, cardiovascular diseases and cancer mortality have largely driven the improvement in the overall unconditional probability of dying, particularly in the Coast and Amazon regions. Peru has adopted several of the Best Buys interventions, Package of Essential NCDs Interventions for Primary Health Care, and HEARTS Technical package for cardiovascular disease management in primary health care [27]. Yet the implementation of these policies and interventions may vary greatly across regions and time; also, how much they have contributed to lessen the burden of cardiovascular diseases has not been quantified.

One policy example is the “Plan Esperanza” (*Hope Plan*), implemented by the Peruvian government since 2012 which allocates resources and set as a priority the prevention, early detection, diagnosis, treatment and palliative care for cancer patients [28]. Until 2014 the epidemiology of cancer in Peru had a mixed profile, with mortality rates due to some neoplasms decreasing and others increasing [29]; recent evidence suggests that the cancer standardized mortality rates have decreased in Peru [30]. To the best of our knowledge there has not been a systematic and consistent analysis of the impact of the “Plan Esperanza”, though the wide and sustained implementation of this program across the country may account for (or some of) our findings regarding the contribution of cancer mortality.

The positive contribution of chronic respiratory diseases and chronic kidney disease to the overall change has been negligible in many regions. The positive contribution of chronic respiratory diseases, we hypothesize, could be explained by the reduction in tuberculosis incidence and the implementation of policies to improve access to clean fuels [31, 32]. The Peruvian government has been implementing “improved kitchens” in places where families were still using solid fuels [32], which are also a risk factor for chronic respiratory diseases. Although this intervention has not been delivered to the whole country yet, it could have played a role to explain our findings regarding chronic respiratory diseases. Tuberculosis is a risk factor for chronic respiratory diseases; if tuberculosis decreases, chronic respiratory diseases would decrease as well, and so would mortality because of chronic respiratory diseases. The positive contribution of chronic kidney diseases may be due to the advancement of policies to secure access to kidney replacement therapy [33], yet there has been limited long-term evaluation of these policies to quantify how they may have affected our findings.

Diabetes, on the other hand, consistently contributed to an increase in the unconditional probability of dying from any selected non-communicable disease. Strong evidence suggests that the prevalence of this condition has increased in the last decades [11, 34], driven by the concomitant obesity burden at the national and sub-national level [10, 35, 36]. An increase in diabetes-associated mortality has also been reported in Peru [37]. In June 2019, the Peruvian government initiated a policy to promote healthy foods that will likely take several years to have benefits. An early evaluation of this policy or its guidelines is beyond the scope of this work; however, our results provide baseline information for future assessments. No other specific population-based policies or interventions have been implemented targeting diabetes, including, for example, providing opportunities for physical activity. In the meantime, people at high risk of diabetes should be identified so that appropriate primary or secondary prevention strategies can be implemented. Because risk stratification tools are available [38], the clinical community could foster their use and inclusion in local guidelines.

One interpretation of our results is that diabetes is moving in the opposite direction of the overall improvement in the unconditional probability of dying; that is, diabetes may be, at best, slowing down the pace to meet the Sustainable Development Goal 3.4. Nonetheless, the other possible explanation is that the other diseases are not improving as fast or as much as needed [39]. From the perspective of health policy or implementation, it may be very challenging to further improve the reduction in other conditions. Peru has made several efforts to implement policies to improve the overall scenario of non-communicable diseases, allocating further resources will require the commitment of policymakers and practitioners. This is particularly relevant in today’s context in which the health system in Peru is investing much resources to control the COVID-19 pandemic, while unfortunately, other diseases may not be receiving as much as attention as before.

The overall change in the probability of dying has mostly improved in the Coast, where there are cities with higher income and better access to healthcare. On the other hand, regions in the Andes, with lower income, had smaller overall reductions. These socio-economic-geographic inequalities have been documented for child health and nutrition [40], though most likely persist and affect morbidity and mortality in adults too. The reductions seen in high-income settings could relate to better access to management and treatment of major cardiovascular disease (e.g., ischaemic heart disease or stroke) and cancer, while diabetes may still be underdiagnosed emerging at the onset of diabetes-related complications. Regarding the unfavourable diabetes contribution across regions, this is consistent with the high obesity prevalence in Peru [35, 41], and with the increasing number of diabetes cases documented in several regions [34, 42, 43]. While policies and resources are much needed to improve health outcomes, they should be carefully thought and wisely allocated thereby contributing to close gaps and to improve health across regions and socio-economic stratum.

We have analysed official mortality records and census data, following a consistent methodology, and building on global evidence to provide specific results for 25 regions in Peru. Moreover, mortality records were adjusted for under-reporting and garbage codes following standard methods [19, 20]. The region-specific estimates will help to guide policies, set priorities and allocate resources so that global targets are met on time or shortly after. The estimates obtained in this work complement the global evidence and strengthen knowledge to assess public health strategic interventions and policies in the effort of reducing premature mortality from NCDs and at national and subnational levels in Peru. Nonetheless, we acknowledge some limitations. To be consistent with the Sustainable Development Goal 3.4 we focused on people aged 30 to 69 years, though we acknowledge that the selected NCDs also represent a large burden of morbidity and mortality among older people [5, 44]. However, people aged 70 years and above account for about 5% of the Peruvian population, thus their inclusion in the analysis would have had little impact. We did not use mortality records and population data for the same exact years; however, this should have not biased the results because the overall population structure would not have substantially changed from one year to the next one. Because of data availability of death rates at the regional level, rather than at the country level available through WHO, we focused on Peru; yet, our findings and conclusions can be informative for other countries with similar geographic and epidemiological profiles, both in Latin American and in other world regions. The quality of mortality records in Peru is not optimal [45], yet it has improved in the last years both in quantity (deaths certificates) and quality (garbage codes). In fact, a work which assessed the Sustainable Development Goal 3.4 in all countries considered that Peru had low vital registration data quality [5]. We reckon this would be a limitation of any work, both local and global, looking at mortality in Peru. Although we thoroughly worked to improve our mortality data inputs [19, 20], interpretation and implementation of our results need to consider the limitations of the original data sources. Our life expectancy estimates are slightly larger than those reported by Peruvian authorities; this could reflect different data sources, data preparation and processing as well as procedures to compute life expectancy. We present life expectancy estimates to compare these with the unconditional probability of dying, and not to replace those provided by national authorities. A shared limitation with other national, regional or global analysis of diabetes-related mortality based on administrative data, is that it relies on awareness of the disease. We believe that unawareness could have underestimated diabetes mortality, thus the unconditional probability of dying estimates may be conservative and suggest a larger burden. Finally, our results are based on purely arithmetical procedures hence the lack of credible intervals.

## Conclusions

Diabetes may prevent Peru from achieving global targets to reduce NCDs mortality. This finding calls to strengthen policies to improve screening, early diagnosis, adequate control and to prevent complications in people with diabetes. Additional population-based policies are needed to reduce the diabetes burden while supporting the seemingly decreasing burden of cardiovascular diseases, cancers, chronic respiratory diseases and chronic kidney disease.

## Supporting information

S1 Table Overall change in the unconditional probability of dying from the selected non-communicable diseases and the contribution of each non-communicable disease by gender and region (main results). All ‘% of the overall change’ add up to 1 (or 100%). The ‘Contribution’ is the multiplication of each ‘% of the overall change’ times the ‘overall change between 2006 and 2016’.
(DOCX)

S2 Table Life expectancy at birth in 2006 and 2016 in Peru and 25 regions by gender. (DOCX)

S1 File Analysis code. (R)
