# Supplementary material for: The contribution of specific non-communicable diseases to the achievement of the Sustainable Development Goal 3.4 in Peru
Source: PLoS One. 2020 Oct 12;15(10):e0240494. doi: 10.1371/journal.pone.0240494 (PMC7549811; doi:10.1371/journal.pone.0240494)
Supplement: S1 Table — All ‘% of the overall change’ add up to 1 (or 100%). The ‘Contribution’ is the multiplication of each ‘% of the overall change’ times the ‘overall change between 2006 and 2016’. (DOCX) [file pone.0240494.s001.docx]

# **S1 Table. Overall change in the unconditional probability of dying from the selected non-communicable diseases and the contribution of each non-communicable disease by gender and region (main results)**

| **Region** | **Sex** | **Change of cardiovascular diseases counterfactual relative to all counterfactuals** | **Change of cancer counterfactual relative to all counterfactuals** | **Change of diabetes counterfactual relative to all counterfactuals** | **Change of chronic respiratory counterfactual relative to all counterfactuals** | **Change of chronic kidney diseases counterfactual relative to all counterfactuals** | **Overall change between 2006 and 2016** | **Contribution - cardiovascular diseases** | **Contribution - cancer** | **Contribution - diabetes** | **Contribution - chronic respiratory** | **Contribution - chronic kidney disease** |
| --- | --- | --- | --- | --- | --- | --- | --- | --- | --- | --- | --- | --- |
| Amazonas | Men | 0.7945 | 0.2727 | -0.0994 | -0.0935 | 0.1257 | -0.2321 | -0.1844 | -0.0633 | 0.0231 | 0.0217 | -0.0292 |
| Amazonas | Women | 0.3498 | 0.8793 | 0.0222 | -0.1641 | -0.0873 | -0.2865 | -0.1002 | -0.2519 | -0.0064 | 0.047 | 0.025 |
| Ancash | Men | 0.5594 | 0.9124 | -0.2644 | -0.2182 | 0.0108 | -0.2267 | -0.1268 | -0.2068 | 0.0599 | 0.0495 | -0.0025 |
| Ancash | Women | 0.5279 | 0.6436 | -0.0868 | -0.048 | -0.0367 | -0.4073 | -0.215 | -0.2621 | 0.0353 | 0.0196 | 0.0149 |
| Apurimac | Men | 8.9133 | 34.2012 | -25.3018 | -24.5028 | 7.6902 | 3.00E-04 | 0.0027 | 0.0104 | -0.0077 | -0.0075 | 0.0023 |
| Apurimac | Women | 0.4148 | -0.5707 | -0.5352 | 0.8083 | 0.8827 | -0.0863 | -0.0358 | 0.0493 | 0.0462 | -0.0698 | -0.0762 |
| Arequipa | Men | 0.608 | 0.1323 | -0.0839 | 0.0868 | 0.2567 | -0.1702 | -0.1035 | -0.0225 | 0.0143 | -0.0148 | -0.0437 |
| Arequipa | Women | 0.3603 | 0.598 | 0.037 | 0.035 | -0.0303 | -0.2051 | -0.0739 | -0.1227 | -0.0076 | -0.0072 | 0.0062 |
| Ayacucho | Men | 0.2859 | 0.6841 | -0.2028 | 0.1477 | 0.085 | -0.2965 | -0.0848 | -0.2029 | 0.0601 | -0.0438 | -0.0252 |
| Ayacucho | Women | 0.5981 | 0.0633 | -0.1468 | 0.1077 | 0.3777 | -0.1436 | -0.0859 | -0.0091 | 0.0211 | -0.0155 | -0.0542 |
| Cajamarca | Men | 0.638 | 0.5959 | -0.3257 | 0.0609 | 0.031 | -0.224 | -0.1429 | -0.1335 | 0.073 | -0.0136 | -0.0069 |
| Cajamarca | Women | 0.358 | 0.4701 | -0.0023 | 0.1226 | 0.0516 | -0.2925 | -0.1047 | -0.1375 | 7.00E-04 | -0.0359 | -0.0151 |
| Callao | Men | 0.5267 | 0.386 | -0.0525 | 0.1704 | -0.0305 | -0.279 | -0.147 | -0.1077 | 0.0147 | -0.0475 | 0.0085 |
| Callao | Women | 0.2214 | 0.6023 | 0.1847 | -0.0983 | 0.0899 | -0.2472 | -0.0547 | -0.1489 | -0.0457 | 0.0243 | -0.0222 |
| Cusco | Men | 0.7677 | 0.4467 | 0.0699 | -0.4176 | 0.1333 | -0.1458 | -0.1119 | -0.0651 | -0.0102 | 0.0609 | -0.0194 |
| Cusco | Women | 0.7266 | 0.4449 | -0.0104 | -0.4072 | 0.2461 | -0.157 | -0.1141 | -0.0698 | 0.0016 | 0.0639 | -0.0386 |
| Huancavelica | Men | 0.0401 | 0.5629 | -0.0805 | 0.3403 | 0.1372 | -0.1324 | -0.0053 | -0.0745 | 0.0107 | -0.0451 | -0.0182 |
| Huancavelica | Women | 0.2604 | 0.4953 | -0.0086 | 0.0154 | 0.2375 | -0.1539 | -0.0401 | -0.0762 | 0.0013 | -0.0024 | -0.0366 |
| Huánuco | Men | 1.5224 | 0.9742 | -1.2494 | 0.0643 | -0.3115 | -0.0388 | -0.0591 | -0.0378 | 0.0485 | -0.0025 | 0.0121 |
| Huánuco | Women | -0.0901 | 0.9857 | -0.0075 | -0.041 | 0.1529 | -0.1881 | 0.0169 | -0.1854 | 0.0014 | 0.0077 | -0.0287 |
| Ica | Men | 0.475 | 0.8541 | 0.022 | -0.3323 | -0.0188 | -0.1816 | -0.0863 | -0.1551 | -0.004 | 0.0603 | 0.0034 |
| Ica | Women | 0.3501 | 0.6596 | 0.0014 | -0.1001 | 0.089 | -0.2397 | -0.0839 | -0.1581 | -3.00E-04 | 0.024 | -0.0213 |
| Junín | Men | 0.716 | -0.1035 | -0.2156 | 0.4245 | 0.1786 | -0.2525 | -0.1808 | 0.0261 | 0.0544 | -0.1072 | -0.0451 |
| Junín | Women | 0.4374 | 0.6117 | -0.4484 | 0.2888 | 0.1106 | -0.1927 | -0.0843 | -0.1179 | 0.0864 | -0.0557 | -0.0213 |
| La Libertad | Men | 0.4685 | 0.6019 | -0.1181 | -0.1071 | 0.1548 | -0.177 | -0.0829 | -0.1066 | 0.0209 | 0.019 | -0.0274 |
| La Libertad | Women | 0.4998 | 0.4453 | -0.1251 | -0.036 | 0.2159 | -0.1829 | -0.0914 | -0.0815 | 0.0229 | 0.0066 | -0.0395 |
| Lambayeque | Men | 0.4344 | 0.609 | -0.1421 | 0.0115 | 0.0871 | -0.2672 | -0.1161 | -0.1627 | 0.038 | -0.0031 | -0.0233 |
| Lambayeque | Women | 0.4131 | 0.5359 | -0.1034 | 0.0198 | 0.1346 | -0.2955 | -0.1221 | -0.1584 | 0.0305 | -0.0059 | -0.0398 |
| Lima | Men | 0.4544 | 0.5614 | -0.1687 | 0.078 | 0.0749 | -0.2205 | -0.1002 | -0.1238 | 0.0372 | -0.0172 | -0.0165 |
| Lima | Women | 0.3514 | 0.5716 | -0.0646 | 0.0608 | 0.0807 | -0.2406 | -0.0845 | -0.1375 | 0.0155 | -0.0146 | -0.0194 |
| Loreto | Men | 0.5006 | 0.1445 | 0.0117 | 0.1991 | 0.144 | -0.3677 | -0.1841 | -0.0531 | -0.0043 | -0.0732 | -0.0529 |
| Loreto | Women | 0.2188 | 0.4391 | 0.117 | 0.121 | 0.1041 | -0.2215 | -0.0485 | -0.0973 | -0.0259 | -0.0268 | -0.0231 |
| Madre de Dios | Men | 0.9347 | -0.1009 | -0.2379 | 0.8821 | -0.478 | -0.2155 | -0.2014 | 0.0217 | 0.0513 | -0.19 | 0.103 |
| Madre de Dios | Women | 0.7196 | 0.2541 | -0.0279 | 0.0806 | -0.0265 | -0.4767 | -0.3431 | -0.1211 | 0.0133 | -0.0384 | 0.0126 |
| Moquegua | Men | 0.4895 | 0.4227 | 0.0285 | 0.2568 | -0.1975 | -0.3719 | -0.182 | -0.1572 | -0.0106 | -0.0955 | 0.0734 |
| Moquegua | Women | 0.3892 | 0.92 | -0.5509 | -0.028 | 0.2697 | -0.2636 | -0.1026 | -0.2425 | 0.1452 | 0.0074 | -0.0711 |
| Pasco | Men | 0.5929 | 0.2361 | -0.2359 | 0.5527 | -0.1458 | -0.1564 | -0.0927 | -0.0369 | 0.0369 | -0.0864 | 0.0228 |
| Pasco | Women | 0.6767 | 0.5809 | -0.2401 | -0.1262 | 0.1086 | -0.2585 | -0.1749 | -0.1502 | 0.0621 | 0.0326 | -0.0281 |
| Piura | Men | 0.5877 | 0.5894 | -0.1685 | 0.0053 | -0.0138 | -0.2429 | -0.1428 | -0.1432 | 0.0409 | -0.0013 | 0.0034 |
| Piura | Women | 0.4868 | 0.5764 | -0.1824 | 0.0013 | 0.1178 | -0.2345 | -0.1142 | -0.1352 | 0.0428 | -3.00E-04 | -0.0276 |
| Puno | Men | 0.4224 | 0.3926 | -0.1874 | 0.3934 | -0.0211 | -0.1753 | -0.074 | -0.0688 | 0.0329 | -0.069 | 0.0037 |
| Puno | Women | 0.6062 | 0.6698 | -1.019 | 0.1073 | 0.6358 | -0.1096 | -0.0665 | -0.0734 | 0.1117 | -0.0118 | -0.0697 |
| San martin | Men | 0.8898 | 0.4865 | -0.513 | -0.0021 | 0.1388 | -0.1243 | -0.1106 | -0.0605 | 0.0638 | 3.00E-04 | -0.0173 |
| San martin | Women | 0.1488 | 0.758 | -0.2463 | 0.1437 | 0.1958 | -0.2312 | -0.0344 | -0.1753 | 0.0569 | -0.0332 | -0.0453 |
| Tacna | Men | 0.2051 | 0.5807 | 0.1151 | -0.2042 | 0.3033 | -0.1036 | -0.0212 | -0.0601 | -0.0119 | 0.0211 | -0.0314 |
| Tacna | Women | 0.1296 | 0.8451 | 0.0665 | -0.2069 | 0.1658 | -0.2416 | -0.0313 | -0.2042 | -0.0161 | 0.05 | -0.0401 |
| Tumbes | Men | 0.4942 | 0.3061 | 0.2145 | 0.0035 | -0.0182 | -0.3566 | -0.1762 | -0.1091 | -0.0765 | -0.0013 | 0.0065 |
| Tumbes | Women | 0.6062 | 0.5445 | 0.0641 | -0.0533 | -0.1615 | -0.4754 | -0.2882 | -0.2588 | -0.0305 | 0.0253 | 0.0768 |
| Ucayali | Men | 0.6144 | 0.5153 | -0.2223 | 0.0441 | 0.0484 | -0.3614 | -0.2221 | -0.1863 | 0.0803 | -0.0159 | -0.0175 |
| Ucayali | Women | -0.5496 | 1.57 | 0.0428 | -0.0597 | -0.0034 | -0.2943 | 0.1617 | -0.462 | -0.0126 | 0.0176 | 0.001 |
| Peru | Men | 0.5286 | 0.5067 | -0.1662 | 0.0594 | 0.0715 | -0.2141 | -0.1132 | -0.1085 | 0.0356 | -0.0127 | -0.0153 |
| Peru | Women | 0.3788 | 0.5862 | -0.0902 | 0.0249 | 0.1003 | -0.2323 | -0.088 | -0.1362 | 0.021 | -0.0058 | -0.0233 |

All ‘% of the overall change’ add up to 1 (or 100%). The ‘Contribution’ is the multiplication of each ‘% of the overall change’ times the ‘overall change between 2006 and 2016’.
