# Supplementary material for: The contribution of specific non-communicable diseases to the achievement of the Sustainable Development Goal 3.4 in Peru
Source: PLoS One. 2020 Oct 12;15(10):e0240494. doi: 10.1371/journal.pone.0240494 (PMC7549811; doi:10.1371/journal.pone.0240494)
Supplement: S2 Table — (DOCX) [file pone.0240494.s002.docx]

# **S2 Table. Life expectancy at birth in 2006 and 2016 in Peru and 25 regions by gender**

| **Region** | **Sex** | **Life expectancy at birth in 2006** | **Life expectancy at birth in 2016** | **Difference in life expectancy** |
| --- | --- | --- | --- | --- |
| Amazonas | Men | 73.91 | 76.95 | 3.04 |
| Amazonas | Women | 79.82 | 84.53 | 4.71 |
| Ancash | Men | 73.43 | 76.64 | 3.21 |
| Ancash | Women | 79.3 | 84.01 | 4.71 |
| Apurimac | Men | 72.06 | 77.91 | 5.86 |
| Apurimac | Women | 77.7 | 84.31 | 6.61 |
| Arequipa | Men | 73.09 | 75.8 | 2.71 |
| Arequipa | Women | 78.75 | 81.63 | 2.87 |
| Ayacucho | Men | 73.6 | 76.81 | 3.21 |
| Ayacucho | Women | 79.33 | 84.25 | 4.92 |
| Cajamarca | Men | 73.47 | 76.6 | 3.13 |
| Cajamarca | Women | 79.33 | 83.98 | 4.66 |
| Callao | Men | 73.55 | 74.33 | 0.78 |
| Callao | Women | 79.29 | 80.05 | 0.76 |
| Cusco | Men | 73.37 | 76.09 | 2.73 |
| Cusco | Women | 78.95 | 83.12 | 4.17 |
| Huancavelica | Men | 73.71 | 76.65 | 2.94 |
| Huancavelica | Women | 79.56 | 83.71 | 4.15 |
| Huánuco | Men | 73.59 | 75.97 | 2.38 |
| Huánuco | Women | 78.35 | 82.2 | 3.85 |
| Ica | Men | 72.71 | 74.69 | 1.99 |
| Ica | Women | 78.03 | 80.38 | 2.35 |
| Junín | Men | 72.92 | 75.92 | 3 |
| Junín | Women | 78.03 | 81.76 | 3.73 |
| La Libertad | Men | 73.34 | 74.84 | 1.5 |
| La Libertad | Women | 79.01 | 81.01 | 1.99 |
| Lambayeque | Men | 73.03 | 74.69 | 1.66 |
| Lambayeque | Women | 78 | 80.35 | 2.35 |
| Lima | Men | 73.29 | 75.8 | 2.51 |
| Lima | Women | 79.12 | 82.34 | 3.23 |
| Loreto | Men | 73.71 | 76.78 | 3.07 |
| Loreto | Women | 78.32 | 84.16 | 5.84 |
| Madre de Dios | Men | 73.51 | 75.15 | 1.64 |
| Madre de Dios | Women |  | 81.89 |  |
| Moquegua | Men | 73.53 | 77.44 | 3.9 |
| Moquegua | Women | 79.74 | 83.18 | 3.43 |
| Pasco | Men | 74.1 | 77.15 | 3.05 |
| Pasco | Women | 80.2 | 84.57 | 4.37 |
| Piura | Men | 72.93 | 75.2 | 2.27 |
| Piura | Women | 78.69 | 81.02 | 2.33 |
| Puno | Men | 72.31 | 76.57 | 4.26 |
| Puno | Women | 77.36 | 82.22 | 4.87 |
| San Martin | Men | 73.66 | 76.77 | 3.1 |
| San Martin | Women | 79.72 | 83.71 | 3.99 |
| Tacna | Men | 73.29 | 75.45 | 2.16 |
| Tacna | Women | 78.06 | 81.91 | 3.85 |
| Tumbes | Men | 73.41 | 77.4 | 3.98 |
| Tumbes | Women | 78.76 | 85.39 | 6.63 |
| Ucayali | Men | 73.76 | 77.23 | 3.47 |
| Ucayali | Women | 80.17 | 84.3 | 4.13 |
| Peru | Men | 73.21 | 75.68 | 2.48 |
| Peru | Women | 78.83 | 82.06 | 3.23 |
